# Transcriptome Analysis Reveals Genes of Flooding-Tolerant and Flooding-Sensitive Rapeseeds Differentially Respond to Flooding at the Germination Stage

**DOI:** 10.3390/plants10040693

**Published:** 2021-04-03

**Authors:** Jijun Li, Sidra Iqbal, Yuting Zhang, Yahui Chen, Zengdong Tan, Usman Ali, Liang Guo

**Affiliations:** 1 National Key Laboratory of Crop Genetic Improvement, Huazhong Agricultural University, Wuhan 430070, China; liy0234@webmail.hzau.edu.cn (J.L.); siqbal@uoswabi.edu.pk (S.I.); ytzhang@webmail.hzau.edu.cn (Y.Z.); cyh2016@webmail.hzau.edu.cn (Y.C.); zdtan@webmail.hzau.edu.cn (Z.T.); usali@student.qau.edu.pk (U.A.); 2 Department of Agriculture, University of Swabi, Swabi 23430, Pakistan

**Keywords:** rapeseed, flooding stress, germination, transcriptome analysis, DEG

## Abstract

Flooding results in significant crop yield losses due to exposure of plants to hypoxic stress. Various studies have reported the effect of flooding stress at seedling establishment or later stages. However, the molecular mechanism prevailing at the germination stage under flooding stress remains enigmatic. The present study highlights the comparative transcriptome analysis in two rapeseed lines, i.e., flooding-tolerant (Santana) and -sensitive (23651) lines under control and 6-h flooding treatments at the germination stage. A total of 1840 up-regulated and 1301 down-regulated genes were shared by both lines in response to flooding. There were 4410 differentially expressed genes (DEGs) with increased expression and 4271 DEGs with reduced expression shared in both control and flooding conditions. Gene ontology (GO) enrichment analysis revealed that “transcription regulation”, “structural constituent of cell wall”, “reactive oxygen species metabolic”, “peroxidase”, oxidoreductase”, and “antioxidant activity” were the common processes in rapeseed flooding response. In addition, the processes such as “hormone-mediated signaling pathway”, “response to organic substance response”, “motor activity”, and “microtubule-based process” are likely to confer rapeseed flooding resistance. Mclust analysis clustered DEGs into nine modules; genes in each module shared similar expression patterns and many of these genes overlapped with the top 20 DEGs in some groups. This work provides a comprehensive insight into gene responses and the regulatory network in rapeseed flooding stress and provides guidelines for probing the underlying molecular mechanisms in flooding resistance.

## 1. Introduction

Of all the environmental challenges that a plant has to face during its growth period, flooding is considered to be of great concern, especially in areas having excessive rainfall or irrigation with poor drainage. Moreover, global warming has also resulted in sea level rise, which is predicted to increase the frequency of oceanic storm surges, heavy precipitation, and flooding events. All these factors are likely to cause significant risk to global agriculture, ultimately posing a challenge for researchers in developing crop varieties capable of growing under such conditions [1,2,3]. Flooding can be subdivided into two phenomena, submergence and waterlogging. Submergence is the drowning of whole plant in water, whereas waterlogging is the flooding of the root zone only. Flooding affects the plant’s ability to absorb oxygen by hindering respiration and replacing gas spaces with excess water leading to low oxygen conditions (hypoxia), which causes a dramatic change in metabolism in order to provide alternate sources of ATP, ultimately jeopardizing plant growth and development [4,5]. Many studies have been conducted on plant response to hypoxia, one such study conducted in *Arabidopsis* identified 49 hypoxia-induced genes encoding transcription factors and regulating anerobic respiration through the glycolytic pathway [6].

A flooding event might be a threat at any stage of crop growth, but germination is one of the most critical developmental phases affected by excess water supply. Seed germination is regulated by various hormones and environmental factors, where adequate oxygen supply is also important before the onset of photosynthetic activity. Flooding during the germination stage is believed to limit mitochondrial respiration, energy metabolism, and the gas diffusion between cells [7]. Restricted aerobic respiration under flooding conditions also leads to outbursts of reactive oxygen species (ROS), which in turn causes reprogramming of gene expression at cellular and molecular levels [8,9,10,11]. As an ROS, hydrogen peroxide (H_2_O_2_) stimulates the expression of Ras homologue gene (Rho)-like small G-proteins, which mediate the activation of *ADH1* expression [12]. Besides ROS, phytohormones also regulate abiotic stress response and seed germination [13,14]. Accumulation of ethylene has been reported as a common phenomenon under flooding conditions. This is attributed to the enhanced expression of a transcription factor *SPEEDY HYPONASTIC GROWTH* [5,15,16]. Ethylene then mediates plant responses to hypoxic conditions by regulating some other transcription factors such as ethylene response factors (ERFs), hypoxia responsive ERF1 (HRE1) and ethylene insensitive 3-like 1a (EIL1a) [17,18]. The members of the group VII ethylene response factors (ERF-VIIs) family are believed to be key players in low oxygen response [19]. RELATED TO APETALA 2.12 (RAP2.12) is a constitutively expressed protein from the ERF family, which stimulates the expression of hypoxia-related genes including alcohol dehydrogenase (ADH) and pyruvate decarboxylase (PDC) under low oxygen conditions [20,21]. Studies have demonstrated that PDC is the main producer of energy under waterlogged conditions in *Arabidopsis*, whereas ADH overexpression enhances the ability of seed germination of transgenic soybean under flooding stress [22,23]. In some plant species, the lactic fermentation pathway also contributes to the waterlogging stress response, where lactate dehydrogenase (LDH) significantly enhances PDC activity under anoxic conditions [24].

Knowledge of genes conferring tolerance to flooding began in the last decade, when studies were conducted to unveil the molecular mechanism behind low oxygen sensing and corresponding signaling [2,3]. Several studies have highlighted plant responses to flooding stress at seedling establishment and later stages [25,26,27,28], but studies on the germination stage are scarce. In general, plants’ response to flooding stress involves shutting down of regular metabolic processes and activation of genes involved in alternate pathways in order to survive and function under variable availability of cellular oxygen. Transcription rates of several genes, associated with oxygen homeostasis, osmoprotectants, and energy metabolism are rapidly adapted in order to scavenge cellular machinery from harmful products of anaerobic metabolism [29]. Transcriptomic analysis has been performed to study seed germination in some crops; examples include legumes, *Arabidopsis*, and cereals [30,31,32].

Flooding is a big concern for rapeseed (*Brassica napus* L.) production in areas with extreme rainfall. Moreover, rapeseed–rice is a routine cultivation practice used for rapeseed production in central China, which can lead to flooding at the germination stage in future years due to the prevailing climatic conditions [33]. Clearly, investigations into potential rapeseed response mechanisms to flooding have received wide attention. Excess soil moisture decreases leaf chlorophyll content and antioxidant enzyme activity, increases the accumulation of toxins and lipid peroxidation levels and decreases the final seed yield and quality [34,35,36]. Transcriptomic analyses have been performed to study rapeseed waterlogging/flooding stress at the seedling stage, while attention has begun to focus on the germination stage [14,37,38,39]; hence, identification of the rapeseed genes responsible for combatting flooding stress at the germination stage is important for rapeseed production. In order to elucidate the gene reprogramming of rapeseed under flooding conditions, we profiled the transcriptome of two rapeseed lines, flooding-tolerant line (Santana) and flooding-sensitive line (23651), under both control and flooding treatment for 6 h at the germination stage. The results provide an insight into the different and complex molecular responses to flooding during germination in tolerant and sensitive rapeseeds.

## 2. Materials and Methods

### 2.1. Plant Material and Flooding Treatment

Flooding-tolerant (Santana) and -sensitive (23651) rapeseed (*Brassica napus* L.) lines were used in this study. Uniform seeds were disinfected with 75% ethyl alcohol for 1 min and then washed with distilled water. Seeds were then placed on moist filter paper under 24 °C until their radicles were about 2–5 mm long. The germinated seeds of both lines were divided into two groups and each group has three replications. One group was supplied with normal water as control, while the other one was filled with double-distilled water in sealed tube (10 mL) as flooding treatment. For evaluation of flooding tolerance, flooding treatment lasted 12 h in the sealed tube [40]. Seeds of both groups were then transplanted into moist vermiculite and cultured for 5 d at 24 °C (light: dark, 16:8 h). Finally, shoot and root length of 4 individuals (control group) and 10 individuals (flooding group) were measured, respectively. For transcriptome profiling, flooding treatment lasted half of the flooding evaluation time (6 h) in the sealed tube, then 20 individuals from each group were harvested at the same time and stored at −80 °C. There were three biological replicates of each group.

### 2.2. RNA Isolation and RNA Library Construction

For RNA-seq, total RNA was extracted from germinated seeds of flooding-tolerant (Santana) and -sensitive (23651) rapeseed lines by RNA prep Pure Plant Kit (TIANGEN, Beijing, China). Total RNA quality and quantity were verified using agarose gel electrophoresis (AGE; gel concentration 1%, voltage 180 V, electrophoresis time 16 min), Nanodrop1000 spectrophotometer (Thermo Fisher Scientific, DE, USA) and Bioanalyzer 2100 (Agilent Technologies, CA, USA). After RNA samples were qualified, mRNA was enriched by magnetic beads with Oligo (dT). Then, fragmentation buffer was added to break mRNA into short fragments, and mRNA was used as template and the first strand of cDNA was synthesized by random hexamers. Next, a chain-specific library was built by dUTP method. Finally, RNA samples were sent to GenoSeq (http://www.genoseq.cn/) (accessed on 31 March 2021) for RNA sequencing using Illumina HiSeq paired-end sequencing.

### 2.3. RNA-Seq Alignment and Differential Expression Analysis

We used FastQC to perform quality control analysis on raw sequencing data [41]. Later, Trimmomatic was used for filtering joints and low quality sequences to obtain the clean data [42]. Hisat2 was then used to align clean sequences to the *Brassica napus* reference genome (http://www.genoscope.cns.fr/brassicanapus/) (accessed on 31 March 2021) and featureCounts was used to calculate the gene expression level as transcripts per kilobase million (TPM) [43,44]. Correlation analysis and principal component analysis (PCA) were performed on gene expression levels and differential expression analysis was performed using DESeq2 package in R to screen differentially expressed genes (DEGs) according to the threshold (|log_2_ (fold change)| > 1 and *padj* < 0.05) [45].

### 2.4. Clustering and Gene Annotation

We used the mclust package in R to perform cluster analysis using the TPM values of differentially expressed genes and list the most significant GO terms in each cluster [46]. In order to annotate the function of DEGs, we aligned *Brassica napus* protein sequence to the *Arabidopsis* database using BLAST tool with E value set to 1e-5 and the coverage > 50% [47].

### 2.5. Quantitative Real-Time PCR Analysis

To validate the RNA-seq data, four DEGs from four different groups were selected for qRT-PCR assay with three biological replicates. All primer pairs were designed using software Oligo7 (DBA Oligo Inc., CO, USA) and shown in Table S1. The RNA samples used for qRT-PCR analyses were the same ones used in the RNA-Seq. RNA from each sample was reverse transcribed using the EasyScript^®^ One-Step gDNA Removal and cDNA Synthesis SuperMix (Beijing Transgen Biotech Co. Ltd., Beijing, China). Quantitative real-time PCR (qRT-PCR) was performed using the PerfectStart^TM^ Green qPCR SuperMix (Beijing Transgen Biotech Co. Ltd., Beijing, China) with the CFX Connect^TM^ Real-Time System (Bio-Rad Laboratories, Inc., CA, USA). The PCR was initiated at 95 °C for 3 min, followed by 45 cycles of 95 °C for 10 s, 60 °C for 10 s, and 72 °C for 10 s. *Actin7* served as internal reference gene to normalize the expression data.

## 3. Results

### 3.1. Flooding-Tolerant and -Sensitive Rapeseed Lines Presented Distinct Phenotypic Differences at the Germination Stage

**Figure 1:**
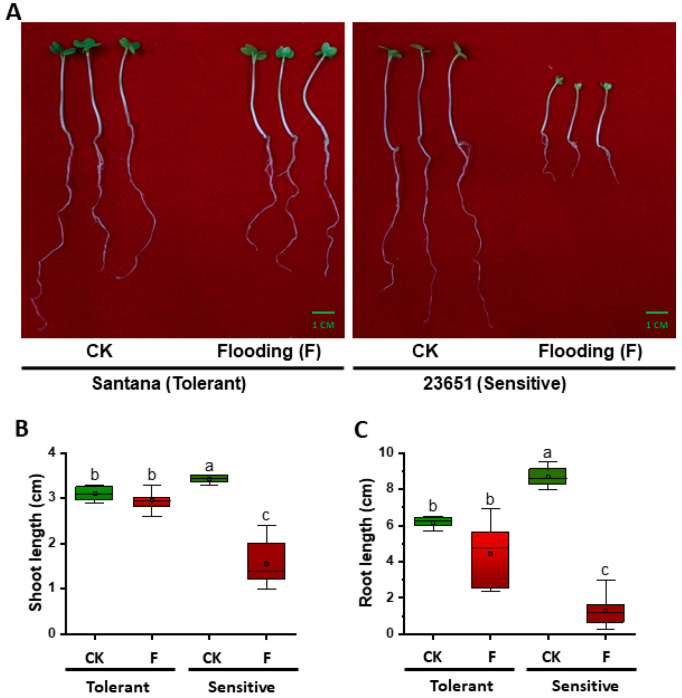
Phenotypic response of tolerant (Santana) and sensitive (23651) rapeseed lines to flooding stress. (**A**) Phenotypes of tolerant and sensitive lines under control treatment and after suffering flooding. Flooding-treated seeds were transplanted into moist vermiculite and cultured for 5 d at 24 °C. Then the growth of seedings was recorded and the pictures were taken. Shoot (**B**) and root length (**C**) of tolerant and sensitive lines under control treatment (CK) and after suffering flooding (F) (*n* = 4 for CK, *n* = 10 for F; medians: 3.10, 2.95, 3.45, and 1.40 for shoot length and 6.25, 84.75, 8.60, and 1.20 for root length). Flooding treatment was repeated three times with consistent results. Letters show significant differences checked by two-way ANOVA test.

In response to flooding, the tolerant line (Santana) was minimally affected during germination, while the sensitive line (23651) had serious differences in its root and shoot length compared to the control (Figure 1A). Under control treatment, root and shoot lengths of the tolerant line (Santana) were 6.25 cm and 3.10 cm, respectively, while in the sensitive line (23651) these were 8.60 cm and 3.45 cm, respectively. Both root and shoot lengths of the sensitive line (23651) were significantly longer than the tolerant line (Santana) under the control condition (Figure 1A,B). When subjected to flooding stress, the root and shoot lengths of tolerant line (Santana) were suppressed to 4.75 cm and 2.95 cm, respectively, while those of the sensitive line (23651) were 1.20 cm and 1.40 cm, respectively. The suppression trend of neither root nor shoot length of tolerant line (Santana) was statistically significant compared to the control, but the opposite was true in the sensitive line (23651) (Figure 1B,C). Moreover, both root and shoot length of the tolerant line (Santana) were longer than the sensitive line (23651) after flooding treatment (Figure 1B,C). 

### 3.2. Hierarchical Clustering of Correlation Matrix Confirmed True Mapping of RNA-Seq Data to Brassica napus Genome

The extracted RNA from three samples of each group was sequenced to examine the changes of gene expression at the germination stage. In order to determine whether the variation of biological replications between samples matched with the expectation of the experimental design, sample correlation was checked by Pearson correlation coefficient. The Pearson correlation coefficients between biological replications of each group were greater than 0.95. Principal component analysis (PCA) was also performed. The PCA analysis successfully distinguished groups from each other and divided biological replications of each group into one cluster, which confirmed that both the flooding treatment and materials were the main factors affecting the gene expression in flooding response (Figure S1A,B).

### 3.3. Flooding Stress Brings Global Gene Expression Changes in Flooding-Tolerant and -Sensitive Rapeseed Lines

**Figure 2:**
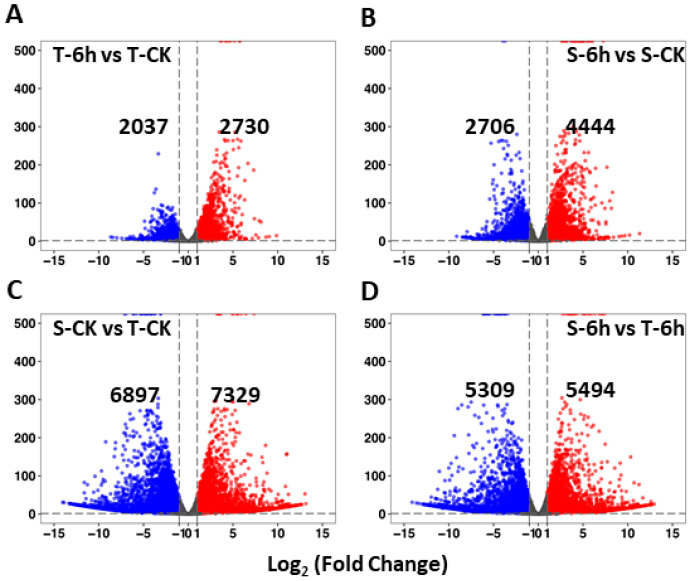
Volcano plots showing the differentially expressed genes (DEGs) between different materials and treatments. (**A**,**B**) DEGs between materials responding to flooding; (**C**,**D**) DEGs between treatments of different materials. CK, control; T, tolerant; S, sensitive.

In order to investigate the nature and dynamics of transcriptomic profiles of flooding-tolerant and -sensitive rapeseed lines under control and 6-h flooding treatments, we identified DEGs with the threshold |log_2_(Fold Change)| > 1 and *padj* < 0.05 (Figure 2A–D). The results were summarized in four groups: (i) tolerant-6 h vs. tolerant-control (T-6h vs. T-CK), (ii) sensitive-6 h vs. sensitive-control (S-6h vs. S-CK), (iii) sensitive-control vs. tolerant-control (S-CK vs. T-CK), and (iv) sensitive-6 h vs. tolerant-6 h (S-6h vs. T-6h). The first two groups were used to find DEGs responding to flooding treatment while the last two groups revealed the specific DEGs between materials under different treatments. A marked difference was observed in the regulation pattern of DEGs in both lines under flooding treatment—the tolerant line had 2037 down-regulated and 2730 up-regulated genes, while the sensitive line had 2706 down-regulated and 4444 up-regulated genes, respectively, when compared with the control (Figure 2A,B). The sensitive line had more up- and down-regulated DEGs than the tolerant line. When differential expression was investigated between flooding-sensitive and -tolerant lines under control and 6-h flooding treatments, 6897 down-regulated and 7329 up-regulated genes were found under control treatment, whereas 5309 and 5494 DEGs (down- and up-regulated) were found under flooding treatment (Figure 2C,D). The sensitive and tolerant lines presented great difference in expression level under control treatment, while the difference reduced under flooding treatment. The complete list of DEGs present in the four groups is available in Table S2.

**Figure 3:**
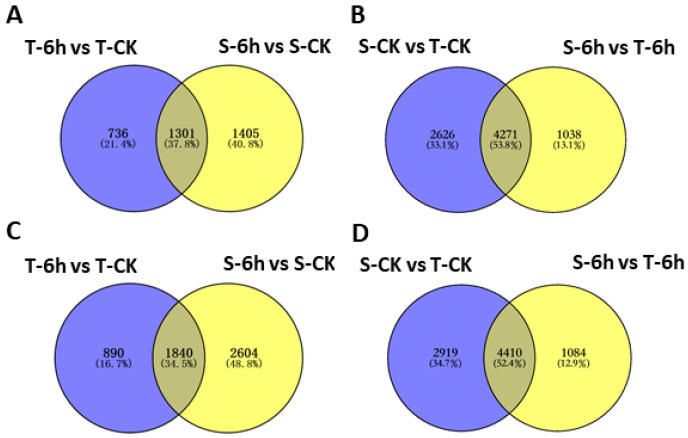
Venn diagrams showing the number of differentially expressed genes (DEGs) between materials and treatments. Down-regulated (**A**) and up-regulated (**C**) genes between materials; down-regulated (**B**) and up-regulated (**D**) genes between treatments. CK, control; T, tolerant; S, sensitive.

Next, we constructed Venn diagram to illustrate the number of commonly shared and specific DEGs by two lines under control and 6-h flooding treatments. By looking at group of genes down-regulated in both tolerant and sensitive lines in respond to flooding, we found 1301 genes having an overlap in their expression, while 736 and 1405 genes were specific to tolerant and sensitive line, respectively, (Figure 3A) whereas 1840 commonly shared DEGs had up-regulated expression, and 890 and 2604 genes were specific to tolerant and sensitive line (Figure 3C). At 6 h of flooding treatment, 4271 and 4410 were commonly shared DEGs having reduced and increased expression in both groups, when compared to control (Figure 3B,D). When compared to tolerant line, there were 2626 down- and 2919 up-regulated genes detected only under control treatment, but 1038 down- and 1084 up-regulated genes detected only under flooding treatment in sensitive line (Figure 3B,D). 

**Figure 4:**
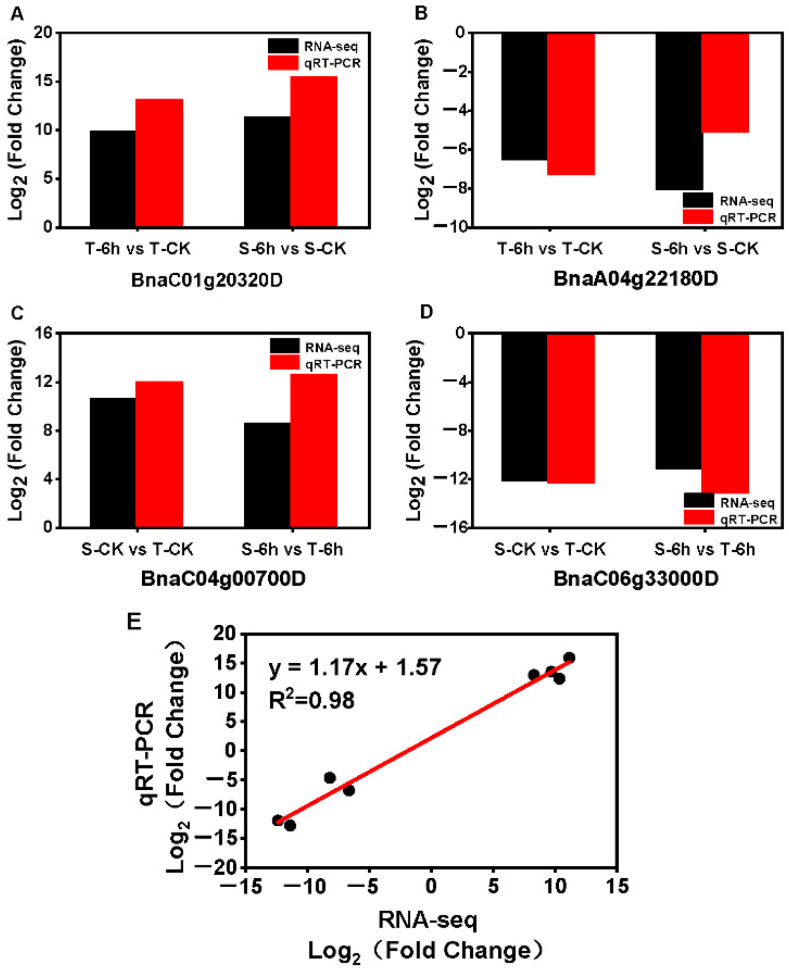
Validation of RNA-seq data by qRT-PCR. (**A**–**D**) The change of expression of 4 representative DEGs between control and flooding were compared based on RNA-seq and qRT-PCR data. Three biological replicates were used for the comparison. (**E**) The expression correlation between the RNA-seq and qRT-PCR. *Actin7* was served as internal reference gene.

To validate the expression levels obtained from RNA-seq, four representative DEGs from the intersection of the four groups were selected for qRT-PCR analysis. The similar trend of relative expression levels of the four DEGs between RNA-seq data and qRT-PCR analyses were observed with significantly positive correlations (R^2^ = 0.98) (Figure 4A–E), suggesting that the results of the RNA-seq were reliable.

### 3.4. Gene Ontology Enrichment Analysis Shows Regulation Pattern of DEGs Responding to Flooding Stress

**Figure 5:**
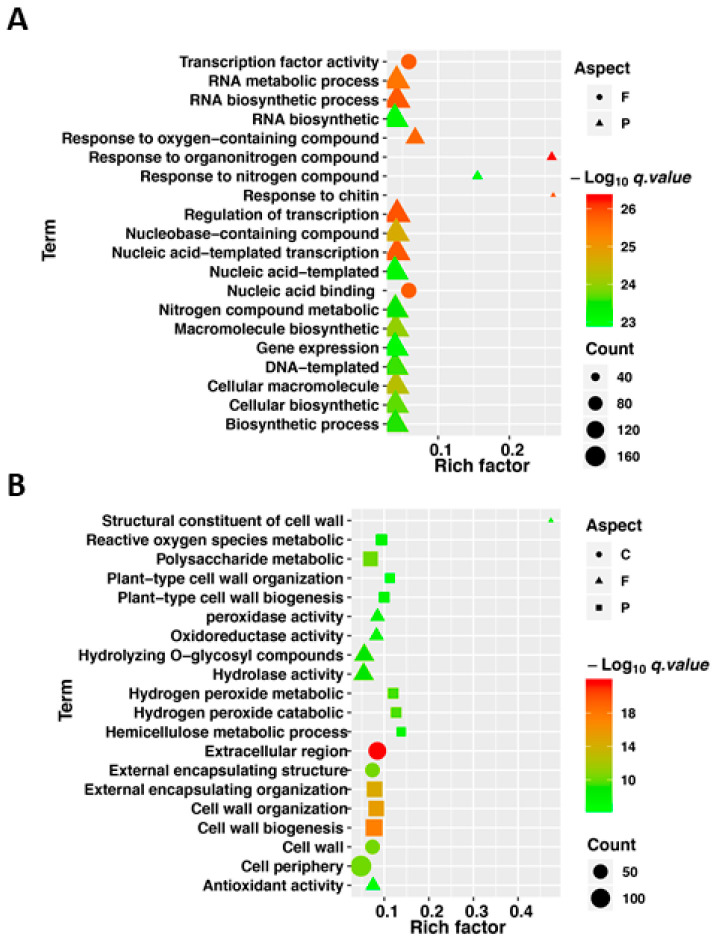
Gene ontology (GO) analyses of up- and down-regulated genes responding to flooding. (**A**) GO enrichment analysis of up-regulated DEGs. (**B**) GO enrichment analysis of down-regulated DEGs. In (**A**) and (**B**), GO enrichment was based on cellular component (C), molecular function (F) and biological process (P). The horizontal axis value shows enrichment factor (*q* < 0.05). All GO terms are displayed as –log_10_ (Fisher’s exact test *q* value).

In order to identify the most significantly shared and unique flooding responsive pathways in tolerant and sensitive lines, we analyzed the top 20 most significantly enriched gene ontology (GO) terms in the enrichment results for up- and down-regulated genes. The GO analysis classified the DEGs into molecular function (F), biological process (P) and cellular compartment (C). The shared up- and down-regulated genes came from the intersection in Figure 3A,C, respectively. The shared up-regulated flooding-response DEGs having significant enrichment were controlling molecular functions (F) including “transcription factor activity” and “nucleic acid binding”, whereas the GO terms related to biological processes (P) such as “response to oxygen containing compounds”, “RNA biosynthesis and metabolic processes”, “transcription regulation”, and “nucleic acid templated transcription” appeared more prominently in the activated genes under flooding stress (Figure 5A). However, down-regulated flooding-response DEGs having significant enrichment were related to molecular function (F) mainly including “structural constituent of cell wall”, “peroxidase activity”, “oxidoreductase activity”, “hydrolase activity”, “antioxidant activity”, related to biological process (P) mainly including “reactive oxygen species metabolic”, “polysaccharide metabolic”, “cell wall organization and biogenesis”, “hydrogen peroxide metabolic and catabolic”, related to cellular compartment (C) mainly including “extracellular region“, and “cell wall organization” (Figure 5B).

These four sets without intersection in Figure 3 were used to explore materials unique flooding-response pathways, which were shown in Figure S2. The tolerant line unique GO terms in flooding stress mainly related to “response to hormone and hormone-mediated signaling pathway”, “protein heterodimerization activity”, “glutamate decarboxylase activity” and “response to organic substance response” (Figure S2A). On the other hand, the unique GO terms differ from materials under control treatment mainly related to “membrane system” and “cell wall macromolecule metabolic process” (Figure S2C), while under flooding condition mainly related to “motor activity, microtubule-based process, cytoskeletal protein binding and tubulin binding”, “regulation of hormone levels”, “cellular respond to auxin stimulus and auxin-activated signaling pathway”, “acting on glycosyl bonds” and “phenylpropanoid biosynthetic process”.

### 3.5. Mclust Analysis Explains the Mechanism of Flooding-Stress Response in Rapeseed

**Figure 6:**
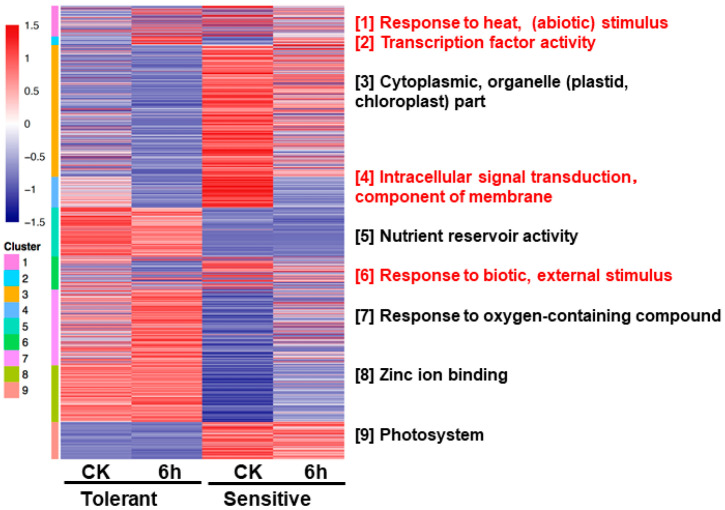
Mclust analysis of DEGs. Heatmap showing the expression pattern of DEGs specific to each group. Nine clusters were generated and named according to the highly significant GO terms found within them (based on *q*-value). Color bars represent enrichment (*q* values) of GO terms of respective DEGs. CK, control.

In order to gain an insight of the differentially regulated biological processes of rapeseed in response to flooding in tolerant and sensitive lines, we performed the mclust analysis of DEGs. Mclust analysis identified 9 different gene clusters, GO enrichment was performed for each gene cluster and the most significant GO term was selected as representative (based on *q* value) (Figure 6). The detail of DEGs (along with their GO ID) specific to each cluster is listed in supplemental information (Table S3).

Mclust modules 1, 2, 4, and 6 showed that expression abundance shared similar change trends in both materials to flooding stress, and were considered to be core modules in flooding response (Figure 6). Mclust module 2 with up-regulated expression genes to flooding stress, mainly corresponded to the significant GO term about “transcription factor activity”; mclust module 4 with down-regulated expression genes to flooding stress, mainly corresponded to the significant GO term about “intracellular signal transduction, component of membrane”; while mclust modules 1 and 6, with a complex response to flooding stress, mainly corresponded to the significant GO terms about “response to heat, (abiotic) stimulus” and “response to biotic, external stimulus”. Mclust modules 3, 5, 7, 8, and 9 showing that expression abundance varied between materials under both conditions, explained the molecular mechanism unique response of materials to flooding (Figure 6). The tolerant line presented higher expression abundance in modules 5, 7, and 8 than the sensitive line, which mainly corresponded to the significant GO terms about “nutrient reservoir activity”, “response to oxygen-containing compound”, and “zinc-ion binding”, respectively. In modules 3 and 9, the sensitive line had higher expression abundance than the tolerant line, which mainly corresponded to the significant GO terms about “cytoplasmic, organelle (plastid, chloroplast) part” and “photosynthesis”, respectively.

### 3.6. Key Genes as Potential Candidates for Flooding Stress

In order to identify representative up-regulated and down-regulated genes for resistance (tolerant and sensitive), and treatment (control and 6-h flooding treatment) level, we analyzed the top 20 DEGs of each group having significant difference in fold change (Table S4).

The shared DEGs were considered to be involved in the common flooding-response mechanism. Among the representative up-regulated DEGs in response to flooding, we identified the RNA transcripts of rapeseed homologues encoding phytosulfokine 2 precursor, DRE-binding protein 2A, heat shock protein 21, and redox responsive transcription factor 1 accumulated at the highest level in both flooding-tolerant and -sensitive rapeseed lines. The other top-most representative genes having significant reduction in expression to flooding included genes encoding annexin 4, (UPF0497), response to low sulfur 2, and laccase 3 (Table 1). When data were investigated for synchronously up-regulated and down-regulated genes conferring rapeseed flooding resistance, the transcriptionally active genes were identified including *ferulic acid 5-hydroxylase 1*, *ribosomal L14p/L23e family protein*, *serine/threonine protein kinase 3*, *D-arabinono-1,4-lactone oxidase family protein*, *photolyase/blue-light receptor 2,* and one *protein of unknown function (DUF3754).* A declining trend was observed in the transcriptional regulation of *NHL domain-containing protein*, *serine hydroxymethyltransferase 2*, *protease-associated (PA) RING/U-box zinc finger family protein,* and *SKU5-similar 5*. The corresponding representative rapeseed genes along with their *Arabidopsis* homologues and fold changes under both treatments are shown in Table 2. In response to flooding, the tolerant line showed unique up-regulated genes mainly including two *FKBP-type peptidyl-prolyl cis-trans isomerase*, two *myb 108*, *jasmonate-zim-domain protein 8*, pyridoxal phosphate *phosphatase-related protein*, and *1-amino-cyclopropane-1-carboxylate synthase 8* (Table S2). Compared to the sensitive line, the tolerant line owned unique up-regulated genes mainly including *OB-fold-like protein*, *SPFH/B* and *7/PHB domain-containing membrane-associated protein*, *carbamoyl phosphate synthetase A*, *TCP-1/cpn60 chaperonin*, *CCCH-type zinc finger family protein*, *alpha-amylase-like 3*, *ribosomal protein S5 domain 2-like superfamily protein*, *choline kinase 1*, *aldolase-type TIM barrel family protein*, *cystathionine beta-synthase (CBS) protein,* and *dehydrin family protein* (Table S4).

## 4. Discussion

Rapeseed first goes through physiological responses to water when the seeds are sown into soil, but excessive water causes soil hypoxia, which leads to germination retardation. Hypoxic/flooding responses of several plant species have also been explored through transcriptome, but the potential mechanism of rapeseed response to flooding remains enigmatic, especially at the germination stage. In the current study, we used tolerant and sensitive lines to elucidate the gene reprogramming in rapeseed under flooding conditions. Compared to one line for transcriptome analysis, comparison of the DEGs between two differential flooding-response lines can verify the universal response mechanism and specifical tolerance processes [14].

Tolerant and sensitive lines both present global gene expression changes to flooding stress (Figure 2). Our results showed a greater number of DEGs were detected in the flooding-sensitive line (23651) than in flooding-tolerant line (Santana) confirming that the flooding-sensitive line was more vulnerable to stress at the molecular level. The phenotypic data (root and shoot length) also explained the consequences of prevailing changes, where root growth was severely constrained. In response to flooding, thousands of genes were up- or down-regulated in both lines (Figure 2). There were many co-regulated genes in response to flooding, which were considered to be responsible for the universal flooding response in rapeseed (Figure 3A,C). On the other hand, we observed that these two lines with different genetic background have quite different gene expression patterns under either normal or flooding conditions. More than 50% of DEGs overlapped between normal and flooding conditions, indicating that tolerant and sensitive lines differ at the physiological level in their responses to flooding (Figure 3B,D). At the same time, we observed large numbers of genes that were uniquely regulated in the tolerant line under flooding conditions, in addition to the overlapped DEGs which comprised an important part of the flooding resistance (Figure 3B,D).

In a previous study, flooding was considered to mainly affect the plant’s ability to absorb oxygen, which causes dramatic change in energy supply, outburst of ROS, and phytohormones, etc. [48,49,50,51,52]. In our study, several GO terms were enriched for the DEGs—these up-regulated DEGs were enriched into terms including “transcription factor activity”, “response to oxygen containing compounds, chitin, nitrogen compound”, and “transcription regulation”, which indicated that transcription-factor-mediated stress response is a core regulatory mechanism to flooding response at the germination stage (Figure 5A). On the other hand, the down-regulated DEGs were enriched into terms including “structural constituent of cell wall, hemicellulose”, “hydrolase activity”, “reactive oxygen species metabolic”, and “peroxidase, oxidoreductase, antioxidant activity”, which reflected that destruction of cell wall structure and intracellular enzymatic reaction activity were drastic affected and the cleaning function of ROS and other toxic substances were seriously impaired by flooding stress (Figure 5B). At the same time, DEGs related to flooding response in the tolerant line were observed to be enriched in “response to hormone and hormone-mediated signaling pathway”, “response to organic substance response” in response to flooding. Meanwhile, in the tolerant line, the DEGs, differently from the sensitive line, were enriched in terms of “motor activity, microtubule-based process”, “regulation of hormone levels”, “acting on glycosyl bonds”, and “phenylpropanoid biosynthetic process”, which indicated that hormone levels and hormone-mediated signaling pathway, biosynthetic process of active substances associated with stress resistance and mobilization of organic substance were key processes conferring rapeseed flooding resistance (Figure S2).

We identified several DEGs having a universal role in multiple environmental cues. Some DEGs having core role in rapeseed flooding response were also identified. Phytosulfokines are 5-amino-acid secreted signaling peptides induced by various fungal elicitors and pathogens and they play a crucial role in organ development, cell differentiation, microbial interactions, and immunity responses [53,54]. The increased root length in tolerant lines might be attributed to enhanced expression of phytosulfokine 2 precursor, as this is mostly expressed in roots and its overexpression was reported to result in lengthier roots coupled with higher frequency of adventitious root formation in *Arabidopsis* [55]. Its vital role in enhancing immunity against fungal infections has been recently reported in rice [56]. The upregulation of DNA replication-related element (DRE) binding protein 2A coupled with enrichment of HSP 21 in both lines under flooding stress highlights the specific role of these genes in flooding tolerance, as the involvement of DRE/DRE-binding factor in osmotic stress response via ABA-independent transcriptional regulation has been previously documented in millet and *Arabidopsis* [57,58,59,60], where it anchors an ethylene- responsive element-binding factor/APETALA2-type (ERF/AP2-type) DNA-binding domain and activates the transcription of multiple downstream genes including heat shock proteins (HSPs) by interacting with DRE-cis element present in their promoter region [59,60,61]. Proteomic analysis performed in soybean roots supported our findings, where abundance of several heat shock proteins in response to flooding was found [62]. Studies have reported diverse roles of HSPs in regulating Rubisco activity under cold temperature stress in sunflower and barley and in chloroplast development even under abnormal growth conditions [63,64,65,66,67]. Under flooding stress, both light and carbon supplies are limited due to the slower diffusion rates in water, hence ATP synthesis is compromised, which causes carbohydrate starvation and disruption in mineral absorption and root hydraulic conductance [68]. We found reduced photosynthesis in both lines under flooding stress with upregulation of *redox-response transcription factor 1 (RRTF1)*. This TF belongs to the family of ethylene-responsive factor (ERF) and plays a vital role in seedling establishment in *Arabidopsis* by regulating redox homeostasis following exposure to photosynthetic perturbations under light and salt stress [69,70]. The special role of ERF-2 in enhancing submergence tolerance and alleviating oxidative damage has been reported in *Arabidopsis* [71].

The unique DEGs between the tolerant and sensitive lines in both environments are likely to confer the rapeseed excellent performance in flooding resistance. Ferulate 5-hydroxylase 1 (FAH1) is responsible for the expression and accumulation of anthocyanin-biosynthesis genes, syringyl lignin deposition in *Arabidopsis,* *and* antioxidant capacity in barley [72,73,74]. Cytosolic calcium level in cell membrane rapidly fluctuates upon sensing of external stimuli. Calcium ions (Ca^2+^) play an important role in stress-signaling cross talk by relaying the stress signals from cell surfaces to effector proteins and initiating downstream responses by regulating interaction of several kinases with target proteins [75]. Some kinases and oxidases are positive regulators of signal transduction, seed germination, amino acid metabolism in response to nutrient deprivation, and plant tolerance to multiple abiotic stresses [76,77,78]. Abundance of receptor protein serine/threonine protein kinase 3 and D-arabinono-1, 4-lactone oxidase family protein under flooding treatment highlights the interaction of these sensors with other proteins resulting in flooding tolerance. Excess water facilitates ROS production and spread of fungal diseases and pathogens that affect root architecture [79,80]. The special role of serine/threonine protein kinase gene against pathogen defense and in enhancing black shank resistance, while elevated tolerance to drought and chilling stress resulted by overexpression of D-arabinono-1,4-lactone oxidase has been reported in tobacco [81,82]. Seed germination is primarily related to the blue wavelength of the spectrum received by cryptochromes. We found increased abundance of DNA photolyase belonging to the blue-light-receptor family. This gene was reported to mediate DNA repair and perception of external light signals [83,84,85]. The detailed mechanism of action of blue light (BL) in regulation of seed dormancy and germination in *Arabidopsis* has been recently reported [86]. Serine hydroxymethyl transferases (SHM) was reported to regulate photorespiration, biosynthetic processes, and salinity tolerance [87,88,89]. Zinc-ion binding is essential step in protein coding. Protease-associated (PA) RING/U-box zinc finger family proteins are involved in protein coding, protein–protein interaction, and trafficking of soluble proteins through subcellular compartments [90]. *SKS5* belongs to a gene family (*SKU5-similar*) related structurally to the multiple-copper oxidases, ascorbate oxidase, and laccase, which playing vital roles in lignin biosynthesis, plant development, and stress responses [91]. What is more, SKU5 and its homologs was reported to be involved in directional root growth by participating in cell wall expansion and synthesis [92,93].

## 5. Conclusions

Comparative transcriptomic profiling employed in this study showed that flooding response in rapeseed at the germination stage involves genes related to transcriptional regulation, zinc ion binding, abiotic stress response, photosynthesis, and cell wall organization. We identified genes corresponding specifically to flooding tolerance and treatment. The discovery of the altered expression of key genes under flooding stress elaborates understanding of the complex and intricate gene regulatory network at the germination stage in rapeseed. The identified candidate genes should be experimentally validated and key genes conferring flooding tolerance of rapeseed could be incorporated in breeding programs for the development of flooding-tolerant/-resistant rapeseed varieties.

## Figures and Tables

**Table 1:** Synchronously up-/ down-regulated genes within top 20 DEGs in flooding response.

Gene ID	*Arabidopsis* Homolog	Description	log2 FC	
			T-6h vs. T-CK	S-6h vs. S-CK
BnaA04g13330D	AT2G22860.1	Phytosulfokine 2 precursor	8.68	9.68
BnaAnng01320D	AT5G05410.1	DRE-binding protein 2A	6.04	7.45
BnaAnng13800D	AT4G27670.1	Heat shock protein 21	9.05	10.20
BnaC01g20320D	AT4G27670.1	Heat shock protein 21	9.87	11.32
BnaC07g45030D	AT4G34410.1	Redox responsive transcription factor 1	7.15	7.84
BnaA04g22180D	AT2G38750.1	Annexin 4	−6.53	−8.09
BnaAnng07630D	AT2G36100.1	Uncharacterized protein family (UPF0497)	−7.43	−7.51
BnaAnng30680D	AT5G24660.1	Response to low sulfur 2	−6.15	−7.96
BnaC04g41010D	AT2G30210.1	Laccase 3	−8.42	−9.13

**Table 2:** Synchronously up-/ down-regulated genes within top 20 DEGs between treatments.

Gene ID	*Arabidopsis* Homolog	Description	log2 FC	
			S-CK vs. T-CK	S-6h vs. T-6h
BnaA01g01400D	AT4G36220.1	Ferulic acid 5-hydroxylase 1	12.64	12.45
BnaAnng00930D	-	-	12.21	11.80
BnaAnng38170D	AT3G04400.1	Ribosomal protein L14p/L23e family protein	12.57	11.87
BnaC02g01280D	AT5G08160.1	Serine/threonine protein kinase 3	12.05	11.92
BnaC04g00700D	AT2G46740.1	D-arabinono-1,4-lactone oxidase family protein	13.06	12.60
BnaC04g38920D	AT3G19340.1	Protein of unknown function (DUF3754)	11.91	12.52
BnaC05g13500D	-	-	11.81	11.66
BnaC06g15890D	-	-	13.15	12.10
BnaCnng57250D	-	-	11.91	11.76
BnaCnng71870D	AT2G47590.1	Photolyase/blue-light receptor 2	12.00	12.69
BnaAnng32430D	-	-	−12.61	−14.10
BnaC06g31340D	AT1G70280.2	NHL domain-containing protein	−12.57	−12.21
BnaC06g32430D	AT5G26780.2	Serine hydroxymethyltransferase 2	−12.70	−12.67
BnaC06g33000D	AT1G71980.1	Protease-associated (PA) RING/U-box zinc finger family protein	−12.46	−13.32
BnaC06g36860D	AT1G76160.1	SKU5-similar 5	−13.12	−12.28
BnaC06g43900D	AT1G73120.1	-	−13.88	−12.63

## Data Availability

The complete RNA-seq data are available at BnTIR (yanglab.hzau.edu.cn/) (accessed on 31 March 2021).

## Supplementary Materials

The following are available online at https://www.mdpi.com/article/10.3390/plants10040693/s1, Figure S1. Gene expression profiles of flooding-tolerant (Santana) and flooding-sensitive (23651) rapeseed lines; Figure S2. Material- specific gene analyses; Table S1. List of primer pairs for qRT-PCT analyses; Table S2. Four groups of differentially expressed genes (DEGs); Table S3. List of nine gene mclust modules; Table S4. Top 20 up- and down-regulated genes of different comparisons.
