# Supplementary material for: Transcriptome Analysis Reveals Genes of Flooding-Tolerant and Flooding-Sensitive Rapeseeds Differentially Respond to Flooding at the Germination Stage
Source: Plants (Basel). 2021 Apr 3;10(4):693. doi: 10.3390/plants10040693 (PMC8065761; doi:10.3390/plants10040693)
Supplement: Supplementary file 1 [file plants-10-00693-s001.zip › Supplementary Files/Figure S.pptx]

## Slide 1
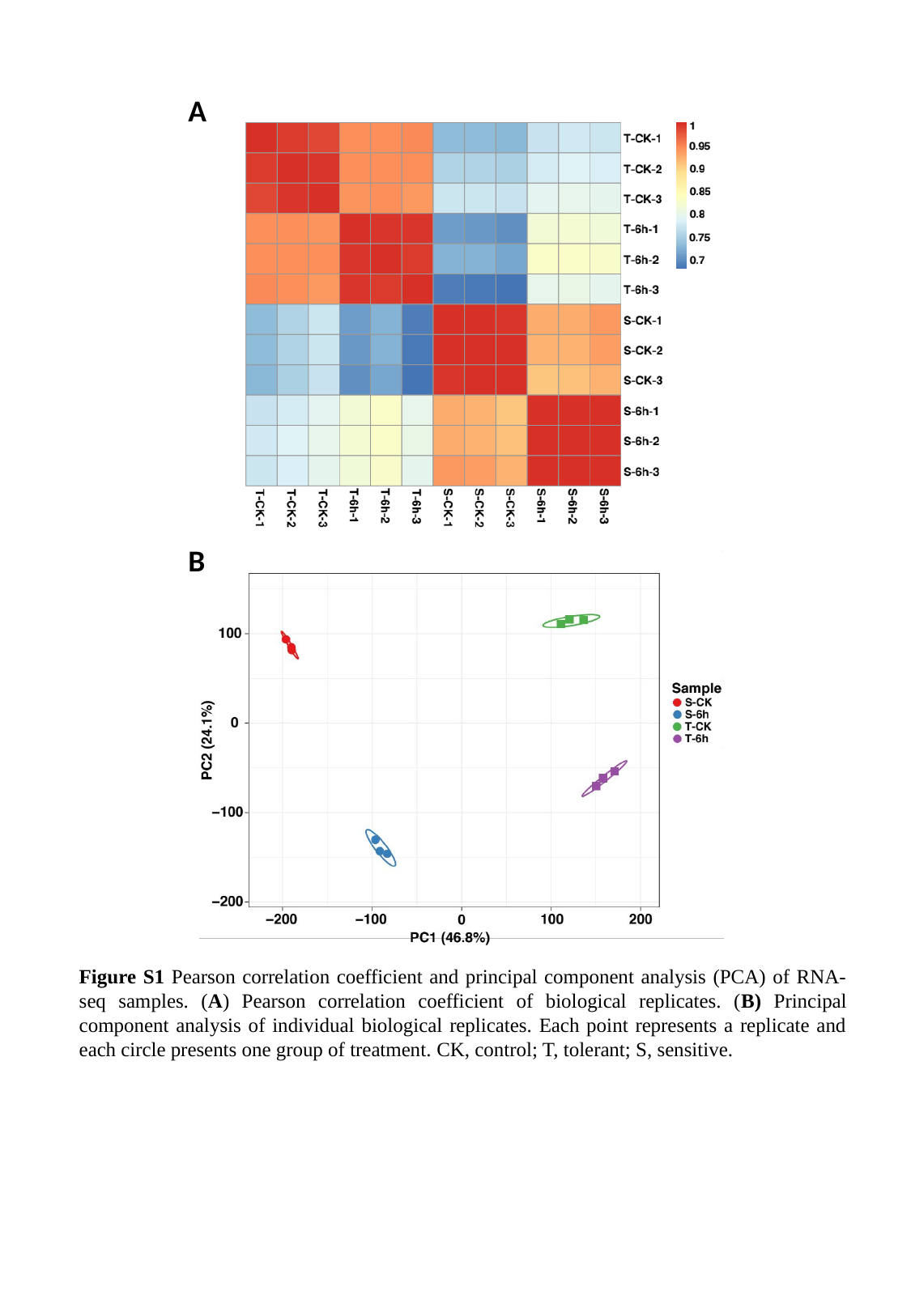

A
B
Figure S1 Pearson correlation coefficient and principal component analysis (PCA) of RNA-seq samples. (A) Pearson correlation coefficient of biological replicates. (B) Principal component analysis of individual biological replicates. Each point represents a replicate and each circle presents one group of treatment. CK, control; T, tolerant; S, sensitive.

## Slide 2
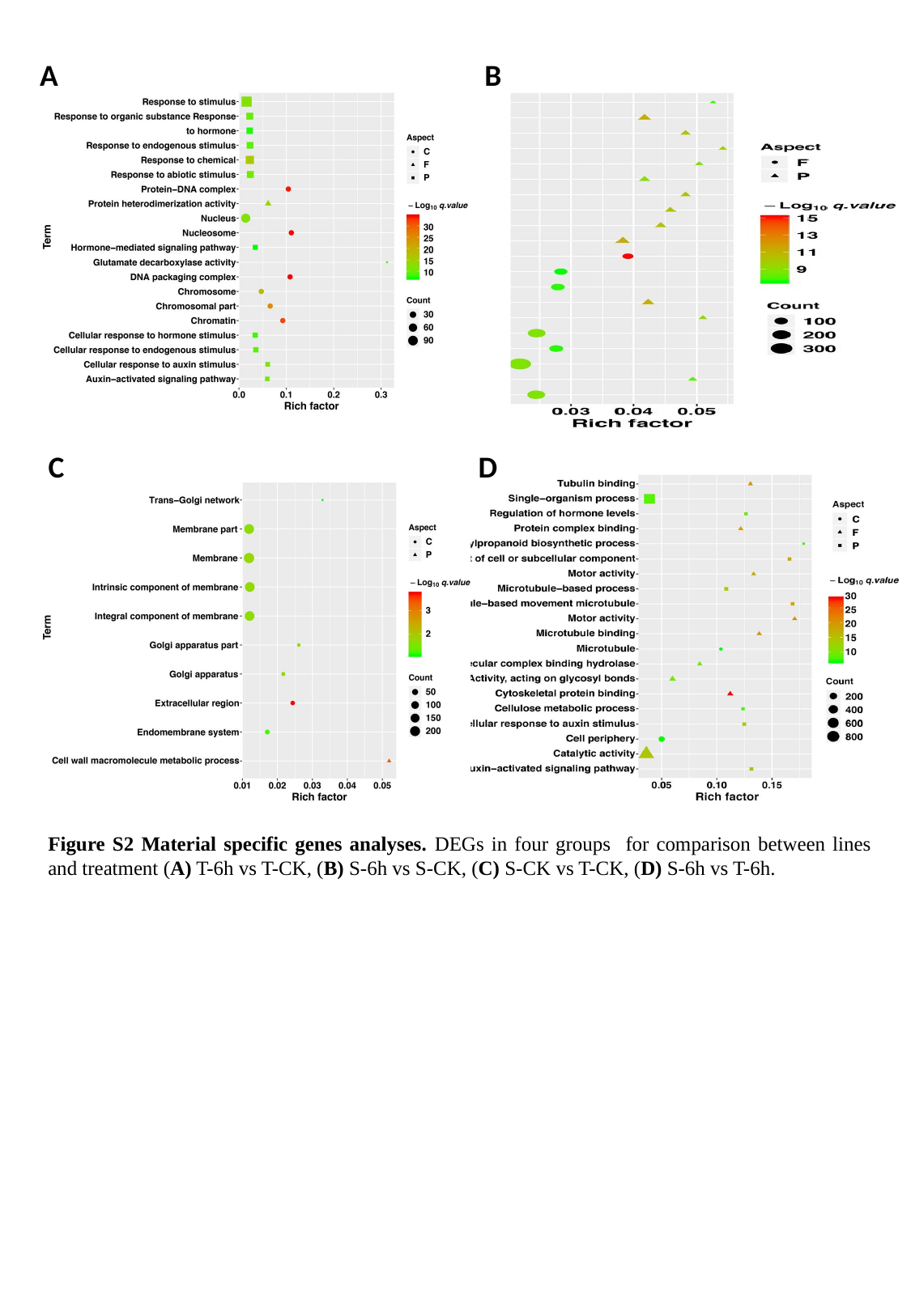

A
B
C
D
Figure S2 Material specific genes analyses. DEGs in four groups for comparison between lines and treatment (A) T-6h vs T-CK, (B) S-6h vs S-CK, (C) S-CK vs T-CK, (D) S-6h vs T-6h.
